# Posttonsillectomy Pain Relief and Wound Healing by Applying Bismuth Iodoform Paraffin Paste (BIPP) to Dissected Tonsillar Beds

**DOI:** 10.1055/s-0043-1777295

**Published:** 2024-03-19

**Authors:** Rahimah Idris, Ramiza Ramza Ramli, Wan NorSyafiqah W Yaacob, Shahid Hassan

**Affiliations:** 1 Department of Otorhinolaryngology, Head and Neck Surgery, Pantai Hospital Laguna Merbok, Bandar Laguna Merbok, Sungai Petani, Kedah, Malaysia; 2 Department of Otorhinolaryngology-Head and Neck Surgery, School of Medical Sciences, Universiti Sains Malaysia, Kota Bharu, Malaysia; 3 International Medical University (IMU) Centre for Education, International Medical University, Bukit Jalil, Kuala Lumpur, Malaysia

**Keywords:** palatine tonsil, tonsillectomy, pain, wound healing, bismuth paste

## Abstract

**Introduction** Tonsillectomy is one of the most common operations performed by otorhinolaryngology surgeons worldwide; however, the insufficient quality of the postoperative pain management and effective posttonsillectomy pain relief remain a clinical dilemma.

**Objective** To evaluate the efficacy of applying bismuth iodine paraffin paste (BIPP) to the dissected fossa as an adjuvant therapy for a better outcome in terms of posttonsillectomy pain management and due to its wound healing properties.

**Methods** The present is a prospective randomized control pilot study with 44 patients aged > 7 years who underwent tonsillectomy. The patients were divided into two groups: the control group and the group that had BIPP applied to the dissected tonsillar fossa. The visual analogue scale score and the post-onsillectomy percentage of tonsillar fossa epithelization were recorded and evaluated.

**Results** Both subjectively and objectively, there a was statistically significant pain-relieving effect in the BIPP group within the first 5 postoperative days ( *p* < 0.05). From postoperative day 3 onward, the dissected area of the tonsillar fossa healed significantly faster in the BIPP group compared with the control group, and it became stable on day 14.

**Conclusion** The topical application of BIPP showed a better pain-relieving effect, it was safe, and hastened wound healing after tonsillectomy.

## Introduction

Even though it has been performed for centuries, tonsillectomy remains controversial, especially in terms of its indications. The procedure is mainly performed for recurrent tonsillitis, obstructive sleep apnea, and as a diagnostic procedure in cases of suspicion of unilateral tonsillar enlargement. Bitar et al. 1 reported that there is enough evidence to conclude that tonsillectomy has no clinically significant negative effect on the immune system. Tonsillectomy is basically an operation to remove the tonsils from their fossae using methods preferred by the surgeon in charge. The methods commonly used are the conventional dissection technique, as well as those assisted by laser, coblation, and harmonic scalpels. Despite the controversy surrounding the indications for tonsillectomy, it is still one of the most common operative procedures performed, and it is regarded as one of the areas of high volume, high-priority practice in Otorhinolaryngology-Head and Neck Surgery (ORL-HNS).

Postoperative pain is a common complication of tonsillectomy. 2 Patients undergoing the procedure mostly complain of throat pain, which leads to odynophagia and dysphagia, thus hindering their recovery. In some cases, postoperative pain following tonsillectomy may be neuropathic in nature, resulting from nerve injury or inflammation. 3 The pain can vary and be quite significant, especially in an adult patient. It is believed that, in adults, the tonsils are usually fibrotic, and their removal, especially with the use of cold instruments, will result in piecemeal removal, which may also result in a wider raw area and significant injury to the surrounding areas. However, there is no clear evidence that the fibrotic nature of tonsils in adults causes pain after tonsillectomy. 2 4 5 6 In a study by Hui and Søvik 7 with pediatric patients, the pain courses varied considerably, and most children had significant pain. Pain after pediatric tonsillectomy should be assessed and treated individually, but, with increasing age, children systematically had lower behavioral pain scores for the same numerical pain score. 7

Other causes of postoperative pain after tonsillectomy include the surgical technique used. 2 4 Choi et al. 8 showed that coblation tonsillectomy induced less pain than electrocautery tonsillectomy in pediatric patients; therefore, they suggested that surgeons choose the coblator as a surgical instrument for tonsillectomy to improve the pediatric postoperative quality of life. 8 Regmi et al. 9 also showed that coblation tonsillectomy significantly reduces the operative time, the intraoperative blood loss, and the postoperative pain.

In general, certain patient factors, such as age, sex, and anxiety, can also contribute to postoperative pain. 10 A systematic review 4 of the literature found that clinicians may underestimate the degree of pain associated with tonsillectomy, leading to the undertreatment of pain.

To manage the pain, analgesics are used postoperatively. Many other methods have also been advocated to improve the management of postoperative pain. 11 12 A randomized clinical trial 6 found that a scheduled oral analgesic dosing regimen was effective in managing postoperative pain in children following tonsillectomy. Another study 5 found that a combination of acetaminophen and ibuprofen was effective in reducing postoperative pain. A prospective, randomized, and double-blinded study found that a combination of intravenous dexamethasone and ketorolac was effective in reducing postoperative pain and opioid consumption. 13

For tonsillectomy, the guideline of the Procedure-Specific Postoperative Pain Management (PROSPECT) group recommends a multimodal approach to pain management, including non-opioid analgesics, opioids, and regional anesthesia. The guideline also recommends the use of dexamethasone and ketorolac for pain management. 4 Bleeding is one of the most common and serious complications of tonsillectomy; it generally occurs in the immediate postoperative period, but it can develop up to 2 weeks after surgery. 14 Posttonsillectomy hemorrhage can be classified as primary or secondary. If bleeding occurs within the first 24 hours after surgery, it is referred to as a “primary hemorrhage.” Secondary hemorrhage occurs after 24 hours. 15 16 The incidence of posttonsillectomy hemorrhage in children and adults varies depending on the study. One study 17 found that the incidence rate of posttonsillectomy hemorrhage in pediatric patients was of 1.83%, with primary hemorrhage accounting for ∼ 33.70% of the cases.

The bleeding, if not diagnosed and treated early and promptly, may lead to a fatal outcome, especially in the younger age groups. According to a study published in 2020, 18 postoperative hemorrhage is a serious complication of tonsillectomy, with secondary bleeding rates affecting up to 0.8% to 3% of patients.

There are few studies on the rate of infection after tonsillectomy. Álvarez Palacios et al. 2 found that the incidence of infection was lower in the coblation group (2.6%) compared with the monopolar dissection group (8.8%). Chen et al. 19 reported that the most common postoperative complications experienced in the first 30 days after surgery were due to infections (58%) and surgical site complications (27%). The most prevalent infections were pneumonia (27% of all complications) and urinary tract infection (27%), and the most common category of surgical site complication was superficial site infection (16%).

### Bismuth Iodoform Paraffin Paste (BIPP)

Bismuth iodoform paraffin paste (BIPP) packed in ribbon gauze is freely available in most, if not all, ORL-HNS departments, because it is commonly used for nasal and aural packing for various reasons.

James Rutherford Morrison, Professor of Surgery at the University of Durham, described the extensive use of BIPP to treat suppurating battlefield wounds during World War I. 20 The wounds were carefully debrided and cleared of any foreign material before the application of BIPP, which was kept in place for days or weeks without being disturbed. Previously, wounds were routinely inspected and repaired following the application of different “antiseptic” solutions. The observed improved antiseptic impact of the BIPP regime could be attributed in part to fewer opportunities for pathogenic bacteria to colonize wounds. 21

N. Athanikar, in an article 22 written in 1996 for the World Intellectual Property Organization (WIPO), states that bismuth compounds are used in the topical treatment of corneal and dermal wounds; they stimulate the release of growth factors in damaged tissues to promote regeneration of epithelial cells making the wound heal at a faster rate. Bismuth compounds are also known to have good antimicrobial activity against several anaerobic organisms; on the other hand, topical preparations of the iodine component of BIPP are also known to have anti-infective properties. 22

Iodoform is a local anesthetic and (clinically) an efficient antiseptic, inhibiting, if not destroying, the microbes the cause putrefaction and pus formation. It has been extensively used in the topical treatment of epithelioma, chancre and chancroid, wounds, ulcers, sores, etc. Regardless of its antimicrobial qualities, if applied liberally to a large raw surface, it may be absorbed in dangerous amounts, resulting in narcotic poisoning symptoms such as increased temperature, quick and feeble heartbeat, collapse, and death. Internally, it is deemed tonic and alterative in small doses.

The paraffin component serves as both a lubricant and a topical anti-inflammatory substance; it decreases the local inflammatory responses of the raw surfaces of the dissected tonsillar fossa. Therefore, considering all the components and properties of BIPP, some centers use it in tonsillectomy by dabbing it on the operated tonsillar bed. It is usually dabbed after the final rinse of the mouth, after both tonsils have been fully removed. It has been used to encourage healing, and it has an analgesic effect after surgery, resulting in decreased throat pain; it also aids in wound epithelization, and shortens the patients' recovery times. Therefore, the present study aims to provide evidence of the effectiveness of BIPP in controlling postoperative pain and promoting wound healing, that is, epithelization of the tonsillar bed in the tonsillectomized patient.

## Methods

### Study Design

The present is a prospective, randomized, experimental, case-control study on the effect of a single application of BIPP on the operated site in selected patients submitted to tonsillectomy at the Penang General Hospital. All patients were operated on by a single surgeon, who is also the researcher, to reduce the operator-dependent effect.

Following approval from the Malaysian Ethics Research Committee (NMRR-09–1091–3685), we selected patients scheduled to undergo tonsillectomies (with or without adenoidectomy) in Penang General Hospital with or without the application of BIPP to the tonsillar fossae. The sample was composed of 44 patients (power of 2) submitted to the procedure (22 patients in each group).

The researchers collected demographic data, such as name, sex, and age, from the subjects. The postoperative pain assessment was performed through the Visual Analog Scale (VAS) whereby the data required (for the pre- and postoperative assessments) were collected from the subjects. The collected data were analyzed using the IBM SPSS Statistics for Windows (IBM Corp., Armonk, NY, United States) software, version 26.0. As for the sampling method, all patients selected for the present study were thoroughly briefed regarding their participation, and they provided informed consent. The patients were randomly selected from operation lists made by by the ORL medical officer, who was not involved in the study. The study and control groups were then randomly selected alternately from the aforementioned lists, regardless of indication for the operation. The tonsillectomies were performed after we ensured that all the patients who had been chosen fulfilled the inclusion and exclusion criteria.

The patients included were physically- and mentally-sound subjects aged > 7 years. For the pediatric patients, consent was provided by a parent or guardian. Patients who were syndromic and had known allergies to components of the BIPP and acetaminophen (paracetamol) were excluded. Tonsillectomies performed for diagnostic purposes or as part of other operations, such as palatopharyngoplasty and glossopharyngeal neurectomy, were also excluded.

### Tonsillectomy Method

After providing informed consent, all patients were operated on using the cold dissection technique by a single surgeon, reducing the bias of the operator-dependent effect. All patients were subjected to the usual preoperative preparations. Routine induction methods were used by the anesthesiologist; after induction and intubation, the patient was placed in the “Rose” position by putting a sandbag on the back of the shoulders, and, using a Boyle-Davis mouth gag, the mouth was opened and the tongue, retracted. The instruments needed were taken from the prepared tonsillectomy set.

Using tonsil-holding forceps, The free end of the tonsils was grasped and stretched medially. An incision was made through the tonsillar capsule at the mucosa of the anterior pillar, the upper pole. Then, by applying the blunt dissector in between the tonsils and their capsules, the tonsils were dissected out of the loose areolar tissues that bind them to the tonsillar bed. The dissection was performed from the upper pole toward the lower pole.

A tonsillar snare was passed around the dissected tonsil pedicle at the lower pole, which was then closed and cut through the pedicle, thus separating the tonsil from its attachment. The dissected tonsillar fossa was packed with small squares of gauze to stop blood from oozing. Then, the prominent bleeding points were stopped using bipolar diathermy. The operative field was washed with normal saline water after hemostasis was completely achieved. Lastly, a ribbon of gauze soaked in BIPP ( Table 1 ) was dabbed and left in contact with each side of the dissected tonsillar fossa for approximately 5 minutes. Then, the gauze was removed, and the operative site was closed.

**Table 1:** Preparation of the bismuth iodoform paraffin paste (BIPP)

Materials:	1. Bismuth subnitrate powder (25 g) 2. Iodoform powder (5 g) 3. Liquid paraffin (5 mL)
Equipment:	1. Weighing scale 2. Glass mortar and pestle 3. Beaker or glass dish 4. Spatula 5. Warm saline 6. Gauze
Instructions:	1. Weigh 25 g of bismuth subnitrate powder and 5 g of iodoform powder using a scale. 2. Add the bismuth subnitrate and iodoform powders to a glass mortar and pestle and grind them together until they are thoroughly mixed. 3. Add 5 mL of liquid paraffin and stir well. 4. Add the bismuth subnitrate and iodoform mixture and stir well until it is thoroughly mixed. 5. Pour the mixture into a glass dish or beaker and soak gauze in it.

**Note:** The BIPP was prepared by the surgeon or the nurses.

### Postoperative Care and Follow-up

The questionnaires and forms regarding the data required for the study (for the pre- and post-operative assessments) were filled out by the patients themselves based on the intensity of the postoperative pain reported using the VAS. Postoperatively, the study and control groups were allowed to take medication as soon as they were able to do so orally. The patients were asked to take acetaminophen only when they needed it for the pain. Total analgesic consumption was recorded daily during hospitalization and then following discharge from the hospital until day 5.

The researcher also examined and recorded the appearance of both tonsils postoperatively, analyzing the color of the tonsillar bed and its stage of epithelization. After discharge, patients and parents were instructed to record pain and analgesic consumption at home. Any occurrence of postoperative complications, such as bleeding or infection, had to be recorded. The filled-out questionnaires will be submitted during the next follow-up visit to the ORL-HNS clinic.

All patients were scheduled for follow-up visits at the ENT clinic under the researcher herself for further assessments. They were examined again on the 3rd, 7th, and 14th postoperative days to monitor the recovery of the tonsillar fossa.

## Results

A total of 44 patients, with ages ranging from 7 to 65 years, were recruited and divided into 2 groups: the study (BIPP) group and the control (non-BIPP) group. None of the patients requested rescue intravenous analgesics, neither did they develop allergic reactions to BIPP or present postoperative bleeding or other complications. None of the patients quit the study, and all attended the follow-up regularly, as instructed.

### Baseline Demographics of the Study Population

The baseline parameters of the study sample were analyzed: the 44 patients examined were 26 children under the age of 12 (pediatric patients) and 18 adults; their mean age was of 19.95 years. In the pediatric group, there were 7 (38.9%) female and 11 (61.1%) male patients; among the adults, there were 14 (53.8%) female and 12 (46.2%) male patients ( Table 2 ).

**Table 2:** Baseline demographics distribution of the study sample

Variables	
1. Patient category: n (%)	
Pediatric	18 (40.9%)
Adult	26 (59.1%)
2. Age in years: median (interquartile range)	14.0 (18.0)
3. Gender: n (%)	
Male	17 (51.5%)
Female	16 (48.5%)
4. BIPP application: n (%)	
No	20 (60.6%)
Yes	13 (39.4%)
5. Preoporative diagnosis: n (%)	
Recurrent tonsillitis	22(66.7%)
Recurrent tonsillitis + adenoids	1 (3.0%)
Peritonsillar abscess	6(18.2%)
Tonsillar hypertrophy + obstructive sleep apnea	4(12.1%)
6. Preoperative grading of tonsillar size: n (%)	
Grade I	4 (12.2%)
Grade II	4(12.1%)
Grade III	12(36.4%)
Grade IV	13 (39.4%)
7. Duration of the operation in minutes: mean(±standard deviation)	46.97(±13.02)

Abbreviation: BIPP, bismuth iodoform paraffin paste.

The two groups were analyzed regarding postoperative pain, frequency and total analgesics consumed postoperatively, appearance of postoperative wound, and the healing process. The 22 patients in each group were further divided into female and male subgroups, which were then separated according to their individual preoperative diagnoses ( Table 3 ). We found no significant correlations involving the variables compared ( *p* > 0.05; Pearson Chi-squared test).

**Table 3:** Visual Analog Scale scores on postoperative days 1, 2 and 3 (D1, D2, D3)

	No pain: n (%)			Mild pain: n (%)			Moderate pain: n (%)			Severe pain: n (%)			Pearson Chi-squared; *p-* value		
Post operative time (D)	D1	D2	D3	D1	D2	D3	D1	D2	D3	D1	D2	D3	D1	D2	D3
1 hour													0.00		
No BIPP	0	0	0	0	3 (13.6)	15 (68.2)	14 (63.3)	19 (84.6)	7 (31.8)	0	0	0			
With BIPP	1 (4.5)	0	0	17 (77.3)	21 (95.5)	16 (72.7)	4 (18.2)	1 (4.5)	0	8 (36.4)	0	0			
3 hours															
No BIPP	0	0	0	0	8 (36.4)	16 (72.7)	14 (63.6)	14 (63.6)	6 (27.3)	8 (36.4)	0	0			
With BIPP	1 (4.5)	0	10 (45.5)	17 (77.3)	22 (100)	12 (54.5)	4 (18.2)	0	0	0	0	0			
6 hours															
No BIPP	0	0	0	0	9 (40.9)	21 (95.5)	16 (72.7)	13 (59.1)	1 (4.5)	6 (27.3)	0	0			
With BIPP	0	0	14 (63.6)	20 (90. 1)	22 (100)	8 (364)	2 (9.1)	0	0	0	0	0			
12 hours															
No BIPP	0	0	1 (4.5)	1 (4.5)	9 (40.9)	20 (90.9)	17 (77.3)	13 (59.1)	1 (4.5)	4 (18.2)	0	0			
With BIPP	0	2 (9.1)	17 (77.3)	21 (95.5)	20 (90.9)	5 (22.7)	1 (4.5)	0	0	0	0	0			
24 hours															
No BIPP	0	0	1 (4.5)	2 (9.1)	11 (50)	20 (90.9)	19 (86.4)	11 (50)	1 (4.5)	1 (4.5)	0	0			
With BIPP	0	3 (13.6)	18 (81.8)	21 (95.5)	19 (86.4)	4 (18.2)	1 (4.5)	0	0	0	0	0			

Abbreviation: BIPP, bismuth iodoform paraffin paste.

### Analysis of Postoperative Pain

The severity of the postoperative pain reported by the groups using the VAS was compared: According to the VAS score the patients were categorized into four groups: 0 = no pain; 1 (VAS score: 1–3) = mild pain; 2 (VAS score: 4–6) = moderate pain; 3 (VAS score: ≥ 7) = severe pain. The assessment of postoperative pain was used as the primary endpoint of the study. The secondary endpoints were the total consumption of analgesics and the total days of analgesic consumption postoperatively. The tertiary endpoints were healing of the wound on the operated tonsillar bed based on the characteristics of the sloughs that had formed and the epithelization of the tonsillar bed.

The VAS score was assessed at 1, 3, 6, 12, and 24 hours postoperatively. The pain scores were recorded from day one (day of the operation) until after the operation had been performed. The data collected was analyzed using the Pearson Chi-Squared test. Table 4 shows a significant difference in the VAS scores on postoperative day 4 between the study and control groups ( *p* = 0.0), as well as a mixed result on day 5. On postoperative days 6 and 7, the the *p* -values were constantly greater than 0.05, indicating the lack of statistical significance regarding differences in pain intensity ( Table 4 ).

**Table 4:** Visual Analog Scale scores on postoperative days 4, 5 and 6 (D4, D5, D6)

	No pain: n (%)			Mild pain: n (%)			Moderate pain: n (%)			Severe pain: n (%)			Pearson Chi-squared; *p-* value		
Post operative time (D)	D4	D5	D6	D4	D5	D6	D4	D5	D6	D4	D5	D6	D4	D5	D6
1 hour															
No BIPP	2 (9.1)	11 (50.0)	19 (86.4)	20 (90.9)	11 (50.0)	3 (13.6)	0	0	0	0	0	0	0.00	0.00	0.116
With BIPP	20 (90.9)	22 (100)	22 (100)	2 (9.1)	0	0	0	0	0	0	0	0			
3 hours															
No BIPP	3 (13.6)	14 (63.6)	19 (86.4)	19 (86.4)	8 (36.4)	3 (13.6)	0	0	0	0	0	0	0.00	0.00	0.116
With BIPP	21 (95.5)	22 (100)	22 (100)	1 (4.5)	0	0	0	0	0	0	0	0			
6 hours															
No BIPP	5 (22.7)	17 (77.3)	19 (86.4)	17 (77.3)	5 (22.7)	3 (13.6)	0	0	0	0	0	0	0.00	0.024	0.116
With BIPP	22 (100)	22 (100)	22 (100)	0	0	0	0	0	0	0	0	0			
12 hours															
No BIPP	9 (40.9)	18 (81.8)	22 (100)	13 (59.1)	4 (18.2)	0	0	0	0	0	0	0	0.00	0.054	> 0.05
With BIPP	22 (100)	22 (100)	22 (100)	0	0	0	0	0	0	0	0	0			
24 hours															
No BIPP	9 (40.9)	18 (81.8)	22 (100)	13 (59.1)	4 (18.2)	0	1 (4.5)	1 (4.5)	0	0	0	0	0.00	0.054	> 0.05
With BIPP	22 (100)	22 (100)	22 (100)	0	0	0	0	0	0	0	0	0			

Abbreviation: BIPP, bismuth iodoform paraffin paste.

**Note:***p* > 0.05 is constant.

### Frequency and Total Amount of Analgesics Taken

The secondary endpoint of the study includes the evaluation of the frequency and total amount of analgesics taken. The analgesic chosen was acetaminophen, which was provided routinely to all patients on a pro re nata (PRN; as needed) basis without exceeding the allowed dose. None of the patients took the rescue analgesic allowed, not even those who presented moderate to severe pain according to the VAS. Table 5 shows a significant difference in the frequency of analgesics taken on the first 3 postoperative days, when both groups were compared using the Mann-Whitney U test ( *p* < 0.05). Indirectly, this indicated that the pain was less severe in the case group compared with the controls. This was further supported by the significant difference in the total analgesics consumed by both groups: the case group took fewer analgesics than the controls, which was statistically significant ( *p* < 0.05) ( Table 6 ).

**Table 5:** Frequency of analgesic (acetaminophen) use

Days	Mean rank		Mann-Whitney U test	*p* -value
	No BIPP	With BIPP		
1	31.57	13.43	42.50	0.000
2	32.59	12.41	20.00	0.000
3	33.00	13.00	32.00	0.000
4	23.50	21.50	220.00	0.152
5	22,00	22.00	231.00	1.000

**Table 6:** Total amount of analgesics (acetaminophen) taken

Medication	Mean rank		Mann-Whitney U test	*p* -value
	No BIPP	With BIPP		
Acetaminophen syrup	12.50	3.50	0.00	0.00
Acetaminophen tablet	21.30	8.63	2.00	0.00

Abbreviation: BIPP, bismuth iodoform paraffin paste.

### Evaluation of Wound Healing of the Dissected Tonsillar Bed

The tertiary endpoint of the present study is an evaluation of wound healing at the operated site. The dissected tonsillar bed was assessed regarding the color (Table 7) and the characteristics of the slough that had formed and its percentage of wound epithelization. Table 8 shows a significant difference in the wound healing process in both groups. There were no documented cases of postoperative bleeding.

**Table 7:** Color of the dissected tonsillar bed

Day	Mean rank		Mann-Whitney U test	*p* -value
	No BIPP	With BIPP		
1	11.50	33.50	0.000	0.000
2	11.50	33.50	0.000	0.000
3	11.50	33.50	0.000	0.000
7	22.50	22.50	242.00	1.000
14	22.50	22.50	242.00	1.000

Abbreviation: BIPP, bismuth iodoform paraffin paste.

**Table 8:** Wound healing of the dissected tonsillar fossa

Day	Grade: n (%)					Chi-squared test; *p* -value
	I	II	III	IV	V	
**Day 1**						> 0.05
No BIPP	22 (100%)	0	0	0	0	
With BIPP	22 (100%)	0	0	0	0	
**Day 2**						> 0.05
No BIPP	22 (100%)	0	0	0	0	
With BIPP	22 (100%)	0	0	0	0	
**Day 3**						0.000
No BIPP	22 (100%)	0	0	0	0	
With BIPP	5 (22.7%)	17 (77.3%)	0	0	0	
**Day 7**						0.000
No BIPP	0	0	21 (95.5%)	1 (4.5%)	0	
With BIPP	0	0	1 (4.5%)	8 (36.4%)	13 (59.1%)	
**Day 14**						> 0.05
No BIPP	0	0	0	0	22 (100%)	
With BIPP	0	0	0	0	22 (100%)	

Abbreviation: BIPP, bismuth iodoform paraffin paste.

## Discussion

Despite advances in anesthesia and surgical technique, postoperative pain after tonsillectomy remains a significant concern. Studies 23 24 assert that 20% to 50% of children submitted to tonsillectomy reported severe pain. The present is the first study to use BIPP as an adjuvant therapy for posttonsillectomy pain relief. It is thought that pain after tonsillectomy is produced by surgical site inflammation, nerve irritation, and pharyngeal spasm. Given that the dissected tonsillar fossa healed as an open wound, it is reasonable to anticipate that local analgesics, antiseptics, and antimicrobial agents such as BIPP may hasten healing and reduce postoperative pain. Furthermore, bismuth compounds are known to trigger the release of growth factors in damaged tissues, promoting epithelial cell regeneration and quicker wound healing. However, it is not possible to keep BIPP in continuous contact with the tonsillar fossa, as in wound dressings. Therefore, BIPP application (dabbed on the dissected tonsillar fossa) is only performed intraoperatively, before the operation ends.

Most of the subjects were adults, with a median age of 14.0, which differs from the worldwide trend, because tonsillectomies have a higher prevalence among children. 25 There was no gender predominance among the 21 (47.7%) female and 23 (52.3%) male patients in the present study. The indications for tonsillectomy were recurrent tonsillitis (66.7%) for most patients, followed by peritonsillar abscess (18.2%), tonsillar hypertrophy with obstructive sleep apnea (OSA; 12.1%), and recurrent tonsillitis with adenoids (3.0%). As for tonsil size, most patients presented grade-III (36.4%) and grade-IV (39.4%), followed by grade-I (12.1%) and grade-II (12.1%). The mean duration of the operation was of 46.97 minutes, with no significant correlations with the preoperative diagnosis and grade of the tonsillar size.

### Analysis of Postoperative Pain

To date, many studies have been conducted to determine the optimal management of posttonsillectomy pain, which remains a clinical dilemma. 2 3 4 6 7 12 14 15 Each treatment proposed has its advantages and disadvantages. Analgesics such as opioids and non-opioid drugs have been advocated, but with known side effects. Opioids may cause sedative effects, nausea, and respiratory depression. Meanwhile, non-opioid analgesics administered as prophylaxis may increase the risk of nausea and bleeding. 26 Acetaminophen is routinely used for pain relief after tonsillectomy, but the effectiveness of this medication when prescribed alone is not clear. 5 Studies 6 have investigated different analgesic protocols to manage pain after tonsillectomy, including the use of metamizole and ibuprofen; however, their results are conflicting, and further research is needed to determine the most effective pain management strategies for tonsillectomy patients.

Topical analgesics are not specifically mentioned in the literature. However, a systematic review 4 of 29 randomized controlled trials found that single-dose analgesics, such as paracetamol, non-steroidal anti-inflammatory drugs (NSAIDs), gabapentinoids, dextromethorphan, and dexamethasone, provide only a weak to moderate benefit on post-tonsillectomy pain on the day of surgery. The authors 4 also recommend a multimodal analgesic approach. A scheduled oral analgesic dosing regimen was also found to be effective to manage pain in children after tonsillectomy. 6

Several studies have been conducted in the past five years to evaluate the potential advantage of local pain control, that is, using local anesthetics and infiltration techniques at the tonsillar fossa to achieve pain relief after tonsillectomy. One study 27 found that postoperative infiltration and packing at the surgical site with bupivacaine significantly reduced the pain associated with tonsillectomy. The pain severity in the bupivacaine group was significantly lower than that in the lidocaine and placebo (normal saline) groups 4, 8, 12, and 16 hours after surgery. 27 Another randomized clinical trial 28 found that honey can be used as an adjunctive regimen after surgery for better pain control. The study 28 found that gargling with honey led to reduced pain following tonsillectomy and that honey lowered the levels of prostaglandin and elevated those of nitric oxide. Many other agents have been tried, such as honey, ketamine, fibrin glue, viscous lignocaine at 2%, and fusafungine. The studies 29 30 31 32 33 showed variable results, with advantages and disadvantages. In the present study, the difference between the case and control groups was statistically significant regarding the VAS scores up to postoperative day 5. The findings strongly suggest that there was a significant reduction in pain after local applications of BIPP on the raw surface of the dissected areas ( Table 3 and Table 4 ). Most patients in both groups started to report great pain relief on the fifth postoperative day. Statistically, from the sixth day onwards, both groups showed no significant difference in pain intensity ( Table 4 ). On the seventh day, the patients were no longer taking analgesics.

It is of not that none of the patients in the case group experienced severe pain, and one patient even stated that they felt no pain in the first three hours after the procedure. This patient felt only mild throat discomfort after the operation. The inability to detect the postoperative pain in the first three hours may be due to the remaining effects of anesthesia.

### Analysis of the Amount and Frequency of Analgesics Taken

The second endpoint of the present study was the analysis of the analgesics taken by the subjects in each group for comparison. The results also showed that the total amount taken by the case group was lower than the amount taken by the control group ( Table 5 ), and this was statistically significant. This result is in line with the study by Junaid et al., 27 who found that the use of local analgesics such as bupivacaine and lidocaine during tonsillectomy reduced postoperative pain. In the present study, this was true for both acetaminophen preparations (syrup or tablet), which showed a Pearson Chi-squared *p* < 0.05. The frequency of analgesic use was lower in the case group, and tis difference was was statistically significant ( Table 6 ). Overall, objectively, this indicates a significant reduction in postoperative pain in the case group. This confirms that the improvement in pain relief in the case group was consistent with the VAS scores reported by the subjects.

### Evaluation of Wound Healing

There is limited recent research available on the wound healing rate after tonsillectomy. Most of the studies available are not recent and focus on other aspects of tonsillectomy, such as pain control and hemorrhage. 6 10 17 19 34 . A review 34 of the literature on wound healing after tonsillectomy was conducted in 2018, and it concluded that the subject had been poorly researched.

Ozlugedik et al. 29 evaluated wound healing based on slough characteristics and the grade of epithelization of the dissected tonsillar bed; these data calculations were considered tertiary endpoints of the study. The wound appearance and epithelization grades of the tonsillar fossa were examined on days 1, 2, 3, 7, and 14. 29 In a study by Akbas et al., 33 the effect of fusafungine on wound healing after pediatric tonsillectomy showed that healing was complete within 10 to 14 days postoperatively.

In the present study, the statistical analysis using a nonparametric two-sample independent *t* -test showed significance ( *p* < 0.05) in terms of wound epithelization in the comparison of the case and control groups. Table 1 shows constant results on postoperative days 1 and 2. In both groups, the operated site was 100% covered with fibrin (grade I) on postoperative day 1. By day 3, there was a significant amount of wound epithelization in the case group, and no epithelization in most of the controls. The earlier wound epithelization in the case group could be due to the anti-inflammatory effect of the paraffin in BIPP, which leads to an earlier reduction in the edema at the operated tonsillar fossae and eventually forms a thin yellowish protective mucosal layer over the operated site. Furthermore, BIPP also has an anti-infective property that may prevent infection at the raw surface of the dissected tonsillar fossa. Early epithelization in the case group could also be attributed to the properties of bismuth compounds, which may stimulate the release of growth factors in the damaged tissues (such as the dissected tonsillar fossae) and promote the regeneration of epithelial cells.

Approaching postoperative day 7, we also observed a significant difference in epithelization grades comparing both groups. More than half of the case group developed epithelization in more than 75% of the dissected areas. All of the patients in both groups were fully recovered by the 14th postoperative day, with a fully-epithelized wound, so no further calculation was needed. With regards to wound appearance, Table 7 shows significant results at postoperative days 1, 2, and 3 because of the color difference in each group. However, the result was not accurate for days 1 and 2, as both initial color differences were due to the yellowish tinge of the BIPP itself, not due to infection or a dirty wound. There were no documented postoperative bleeding complications in any of the cases operated; this could be because the bleeding area was immediately secured following tonsillectomy, and the BIPP dapping was applied adequately. On postoperative days 7 and 14, no significant difference was observed. After analyzing all the results, we found that topical applications of BIPP onto the dissected tonsillar fossa significantly improved wound healing after tonsillectomy.

In terms of BIPP-related complications, direct bismuth aspiration has been reported 35 to cause pulmonary complications in laboratory animals, but no clinical correlation in humans had been previously described until Murray et al. 35 reported two pediatric cases of bismuth aspiration leading to respiratory difficulties after tonsillectomy and adenoidectomy; neither child's respiratory condition necessitated airway intubation. With that in mind, the application of BIPP, like all topical medications, must be performed with caution and following proper guidelines to minimize potential risks.

## Conclusions

Despite advances in anesthesia and surgical technique, pain after tonsillectomy remains a significant concern. The application of BIPP to the tonsillar fossa after tonsillectomy resulted in lower pain intensity throughout the postoperative period, as evidenced by the use of less potent analgesics by the case group compared with the control group. The application of BIPP hastens the wound healing process promotes better and faster epithelization of the operated site.
